# Key Role of STAT4 Deficiency in the Hematopoietic Compartment in Insulin Resistance and Adipose Tissue Inflammation

**DOI:** 10.1155/2017/5420718

**Published:** 2017-03-16

**Authors:** Anca D. Dobrian, Kaiwen Ma, Lindsey M. Glenn, Margaret A. Hatcher, Bronson A. Haynes, Eric J. Lehrer, Mark H. Kaplan, Jerry L. Nadler

**Affiliations:** ^1^Department of Physiological Sciences, Strelitz Diabetes Center, Eastern Virginia Medical School, Norfolk, VA, USA; ^2^Department of Internal Medicine, Strelitz Diabetes Center, Eastern Virginia Medical School, Norfolk, VA, USA; ^3^Department of Pediatrics, Indiana University School of Medicine, Indianapolis, IN, USA

## Abstract

Visceral adipose tissue (AT) inflammation is linked to the complications of obesity, including insulin resistance (IR) and type 2 diabetes. Recent data from our lab showed that germline deficiency in STAT4 reduces inflammation and improves IR in obese mice. The objective of this study was to determine the contribution of selective STAT4 deficiency in subsets of hematopoietic cells to IR and AT inflammation. To determine the contribution of hematopoietic lineage, we sublethally irradiated* Stat4*^−/−^*C57Bl6* mice and reconstituted them with bone marrow cells (BMC) from* Stat4*^*+/+*^*C57Bl6* congenic donors. We also established the contribution of selective STAT4 deficiency in CD4+ or CD8+ T cells using adoptive transfer in* Rag1−/−* mice. All mice received a HFD for 15 weeks (*n* = 7–12 mice/group). BMC that expressed STAT4 induced increases in glucose intolerance and IR compared to STAT4-deficient cells. Also, AT inflammation was increased and the numbers of CD8+ cells infiltrating AT were higher in mice with STAT4 expressing BMC. Studies in* Rag1−/−* mice further confirmed the prominent role of CD8+ cells expressing STAT4 in insulin resistance and AT and islet inflammation. Collectively our results show specific and dominant contribution of STAT4 in the hematopoietic compartment to metabolic health and inflammation in diet-induced obesity.

## 1. Introduction

Inflammation and activation of the immune system in obesity are emerging as key contributors associated with type 2 diabetes and cardiovascular disease. Activation of various inflammatory pathways in visceral adipose tissue (AT) in obesity was recently identified as an early indicator of insulin resistance and type 2 diabetes and as a contributor to disease susceptibility and progression [1].

AT is a heterogeneous tissue and multiple cell types become dysfunctional with increased adipose mass. Proinflammatory cytokine and chemokine production is increased in hypertrophied adipocytes, in activated macrophages, and in T cell subsets infiltrating adipose tissue in obesity [2]. In turn, inflammatory mediators produced in AT contribute to adipocyte metabolic dysfunction resulting in increased lipolysis and impaired glucose uptake, which can induce pancreatic beta cell dysfunction, insulin resistance, and atherosclerosis [3–6].

One of the pathways activated in chronic AT inflammation is the IL12/STAT4 pathway [7, 8]. STATs are downstream of the Jak/Tyk tyrosine kinases and act as transcription factors inducing expression of genes involved in proliferation and differentiation of various hematopoietic and nonhematopoietic cells [9, 10]. The role of STAT4 was best characterized as one of the drivers of Th1 polarization and NK cell activation [11, 12]. However, STAT4 is also present in macrophages and dendritic cells and mediates production of IFN*γ* or other proinflammatory cytokines in response to IL12/IL18 or adiponectin [13–15].

Our group recently reported expression of STAT4 in adipocytes and increased STAT4 activation in visceral adipose tissue in rodent obesity [16, 17]. Also, we reported that STAT4 global deletion reduces insulin resistance and adipose tissue inflammation in obesity in part by reducing adipocyte hypertrophy, infiltration of immune cells, by promoting M2-biased macrophage polarization, and by improving insulin signaling in adipose tissue [17].

In this paper we sought to more clearly define the role of selective STAT4-deficiency in subsets of hematopoietic cells versus adipocytes. We found that* Stat4−/−* deficiency in bone marrow cells rendered the recipient mice less insulin resistant and glucose intolerant. We identified increased numbers of CD8+ cells, increased numbers of Mac-2 positive crown-like structures, and adipocyte hypertrophy in visceral adipose tissue of* Stat4*+/+ hematopoietic recipients, despite STAT4 deficiency in adipocytes.

To further establish the role of STAT4-deficiency in T cell subsets, we adoptively transferred STAT4-deficient CD4+ or CD8+ cells in* Rag1−/−* mice.* Rag1* null mice are lacking mature T, NKT, and B cells [18], have functional antigen presenting cells and, similarly to* C57Bl6/J* mice, become obese and insulin resistant in response to high fat diet [19]. In this study* Rag1−/−* mice were used for selective reconstitution with STAT4-deficient or STAT4-sufficient T cell subsets and challenged with high dietary fat. We found that glucose tolerance was improved only in* Stat4−/−* CD8+ recipients on high fat diet and this was likely due to an insulinotropic effect.

These studies highlight the importance of STAT4 in subsets of hematopoietic cells and its contribution to inflammation and insulin resistance in obesity.

## 2. Materials and Methods

### 2.1. Animals and In Vivo Protocols

All procedures involving animals were approved by the IACUC of Eastern Virginia Medical School. Male C57Bl/6J mice and Rag1−/− mice were purchased from Jackson Laboratories (Bar Harbor, ME).* Stat4−/−* C57Bl/6J mice (gift from Dr. Mark Kaplan from Indiana University) and littermate controls were bred in the EVMS facility. We applied 2 in vivo protocols (Supplemental Figure 1 in Supplementary Material available online at https://doi.org/10.1155/2017/5420718). In* Protocol 1*, male* Stat4−/−* C57Bl/6J and littermate controls, 8 weeks old, were sublethally irradiated using 2 irradiation regimens of 6 Gy each with a 4-hour interval between irradiation processes (orthovoltage X-ray machine). Immediately following irradiation mice were transplanted via tail vein injection with 2.5 × 10^7^ bone marrow cells collected from tibial and femoral bones of either* Stat4−/−* C57Bl/6J mice or C57Bl/6J mice, 8–10 weeks old. Mice were allowed to fully recover for 4 weeks and were subsequently switched to a high fat diet (60% kcal fat, Research Diets, New Brunswick, NJ) for 15 additional weeks. All of the mice were between 27-28 weeks of age at euthanasia (*n* = 8/group). In* Protocol 2*, male* Rag1−/−* mice, 10 weeks of age, were adoptively transferred with either CD4+ or CD8+ splenocytes isolated from age and gender matched* Stat4−/−* C57Bl/6J or C57Bl/6J mice (*n* = 6–12/group) (Supplemental Figure 1). Splenocytes were enriched in either CD4+ or CD8+ T cells by positive selection using magnetic immunoseparation (StemCell Technologies, Vancouver, Canada). After enrichment, the purity was typically >95%. 5 × 10^6^ cells were injected via tail vein. Some of the* Rag1−/−* mice were injected with saline and served as controls. One week following the adoptive transfer, all of the mice were placed on HFD for 15 weeks.

Mice were housed in a pathogen-free facility and individually caged and food and water were provided ad libitum throughout the experiment. Body weight and food intake were measured weekly.

### 2.2. Metabolic Measurements

The intraperitoneal glucose and insulin tolerance tests were performed at the end of the chronic dietary challenge, as previously described [20, 21]. Plasma insulin was measured using a commercially available ELISA kit from Mercodia (Uppsala, Sweden), according to manufacturer's instructions. Plasma glucose was measured using a kit from Cayman Chemicals (Ann Arbor, MI). Free fatty acids, triacylglycerols, and cholesterol were measured with a kit from Wako (Richmond, VA).

### 2.3. Western Blotting for In Vivo and In Vitro Insulin Signaling

Prior to euthanasia, mice were injected i.p. with a bolus of 10 U/kg insulin or with saline and after 10 minutes adipose tissue, liver, and muscle were harvested and homogenized on ice in RIPA buffer with protease/phosphatase inhibitors. To determine the activation of the insulin signaling pathway, insulin receptor (IR), insulin receptor substrate-1 (IRS-1), and protein kinase B (Akt) phosphorylation and total protein expression were measured by western blotting using the following antibodies: pIR (Y972, Abcam, 1 : 500 dil); IRbeta (Cell Signalling, 1 : 1,000 dil); pAkt (Ser473, Cell Signalling, 1 : 1,000 dil); Akt (Cell Signalling, 1 : 1,000 dil); pIRS-1 (Y896, Invitrogen, 1 : 500 dil); IRS-1 (Cell Signalling, 1 : 500 dil). *β*-Actin (Santa Cruz, 1 : 2,000 dil) was used to normalize in some cases for protein loading. Activation of the same proteins was also measured by western blotting in isolated adipocytes obtained from epididymal adipose tissue following collagenase digestion. Adipocytes were analyzed in the basal state or following stimulation with 5 nM insulin for 30 minutes.

### 2.4. Pancreatic Islet Isolation and In Vitro Glucose Stimulated Insulin Secretion (GSIS)

Pancreatic islets were isolated by pancreatic perfusion with collagenase P (Roche Applied Science) through the common bile duct as previously described [21]. For GSIS islets were incubated in Krebs Ringer Bicarbonate Buffer (KRHB) supplemented with 3 mM glucose for 1 hour followed by an additional one-hour incubation in 28 mM glucose, as previously described [21]. The supernatant was collected after each of the treatments and insulin was measured by an ELISA method (Mercodia, Uppsala, Sweden).

### 2.5. Adipose Tissue Morphometry

Epididymal tissue was formalin fixed and paraffin-embedded and the sections were stained with hematoxylin and eosin. Three representative images were obtained per section, for a total of 6 tissue sections/mouse. Four mice/experimental group were randomly selected and analyzed. Images were taken using a Zeiss Plan Apochromat 20x objective and adipocyte area and number were obtained using the ImageJ software (NIH, Bethesda, MD).

### 2.6. Immunofluorescence Staining

Paraffin-embedded adipose tissue sections were treated for antigen retrieval and incubated with primary antibodies for STAT4 (goat anti-mouse, Santa Cruz, CA, 1 : 100 dil) and Mac-2 (rabbit anti-mouse, Abcam, 1 : 100 dil). Nuclei were stained using DAPI (1 : 1,00 dil). Pictures were taken using Zeiss Plan Apochromat 20x or 40x objectives.

### 2.7. Adipocyte and Stromal Vascular Fraction Preparation

Samples of epididymal adipose tissue (0.1–0.3 g) were digested with collagenase as described before with minor modifications [22, 23]. The floating adipocytes were collected and washed and the infranatant was removed and centrifuged at 500 ×g, for 5 minutes to pellet the stromal vascular fraction (SVF).

### 2.8. Flow Cytometry

Counted SVF cells were incubated for 30 minutes, at room temperature with one of the following combinations fluorophore-conjugated primary antibodies:* Cocktail 1* (for macrophage phenotyping): CD11b-Pacific Blue, CD45-PerCP, and Cd11c-PE F4/80-Alexa 647;* Cocktail 2* (for T cell phenotyping): CD3-Pacific Orange, CD4-APC, CD8-FITC, and CD45-PerCP. All of the antibodies were from BD Pharmingen (San Jose, CA) or from BioLegend (San Diego, CA). Cells were analyzed on a BD upgraded FACS Caliber Flow Cytometer (8 colors) using FlowJo software (Tree Star Inc., Ashland, OR). Also spleens were collected; single cell suspensions were prepared by gentle mechanical disruption and were stained similarly to protocols described for SVF.

### 2.9. Real-Time PCR

RNA from total adipose tissue, pancreatic islets, adipocytes, or SVF was extracted and reverse transcribed as previously described [24]. Real-time PCR was performed using Taqman probes from Applied Biosystems (Carlsbad, CA). *β*-Actin was used to normalize the data. Results were expressed either as 1/ΔCt or as fold change by the 2^−ΔΔCT^ method using the wild-type mice as a control group. The multiplex gene array PCR for adipocytes was performed using the Mouse Chemokine and Chemokine RT^2^ Profiler PCR Array (PAMM150Z) from SA Bioscience (Carlsbad, CA).

### 2.10. Statistical Analysis

Statistical analysis was performed using GraphPad Prism Software (GraphPad Software Inc., La Jolla, CA). Student's *t*-test unpaired analysis was used for all data comparisons between two groups (Protocols 1 and 2) and one-way ANOVA was used for comparisons between 3 or more groups (Protocol 2). Data were expressed as mean ± SD and the null hypothesis was rejected for a *p* value < 0.05.

## 3. Results

### 3.1. STAT4-Deficiency in the Hematopoietic Compartment Improves Glucose Tolerance and Insulin Sensitivity in Diet-Induced Obesity

**Figure 1:**
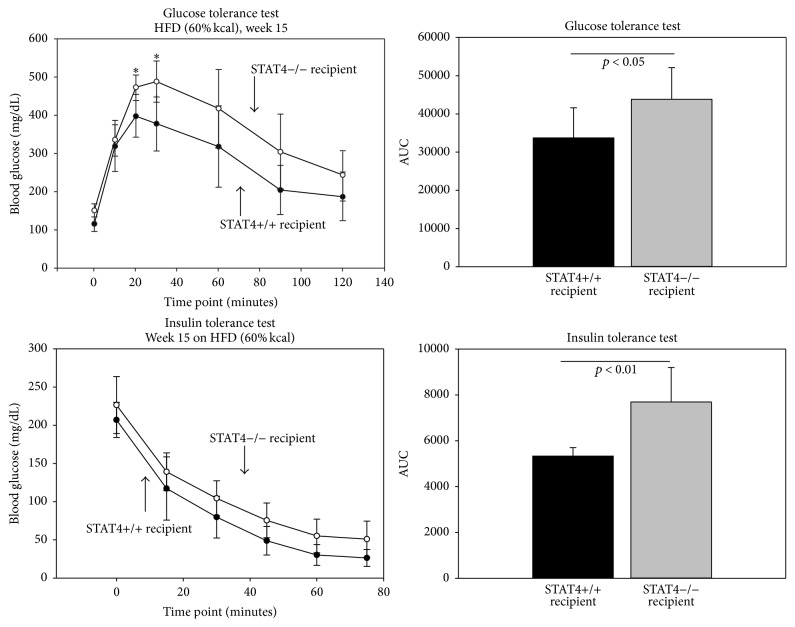
Intraperitoneal glucose tolerance test (GTT) and insulin tolerance test (ITT) were performed after 15 weeks of HFD. For both the ITT and the GTT, STAT4*−/−* recipients that received STAT4+/+ bone marrow cells had significantly higher area under curve (AUC) compared to STAT4+/+ recipients that received STAT4*−/−* cells. Data represents average ± SD from 7 mice/group. The null hypothesis was rejected for a *p* value < 0.05; ^*∗*^*p* < 0.05.

STAT4 is expressed predominantly by cells in the myeloid and lymphoid compartments, but also in solid organ cells including adipocytes. To determine the selective role of STAT4 deficiency in the myeloid compartment, C57Bl/6J mice were transplanted with bone marrow cells (BMC) from STAT4-deficient mice (*Stat4*+/+ recipients) and compared to STAT4-deficient mice transplanted with (BMC) from wild-type littermates (*Stat4−/−* recipients) (Figure S1). The degree of chimerism determined based on presence of STAT4 gene in BMCs, blood, and spleen showed >98% of the genetic signature of the donor cells in blood and BMC and >85% in spleen (Figure S2). There were no significant differences between body weights and fasting plasma cholesterol, fatty acids, and triglycerides between the two mice groups (Table 1). However,* Stat4−/−* recipient mice that received wild-type bone marrow had ~25% higher fasting plasma glucose compared to* Stat4*+/+ recipient mice that received* Stat4−/−* hematopoietic cells (Table 1). In addition,* Stat4*+/+ recipient mice that received* Stat4−/−* BMCs showed significantly improved glucose tolerance as determined by an intraperitoneal glucose tolerance test and the area under curve (Figure 1). Also, the insulin tolerance test showed improved insulin sensitivity in the mice that received* Stat4−/−* BMC (*Stat4*+/+ recipients) (Figure 1). This result indicates a key role of STAT4 deficiency in the hematopoietic compartment in the improved metabolic phenotype.

### 3.2. Recipient Mice of STAT4-Deficient BMCs Show Reduced Visceral Adipose Tissue Inflammation and Lymphocyte Infiltration in Response to High Fat Diet

**Figure 2:**
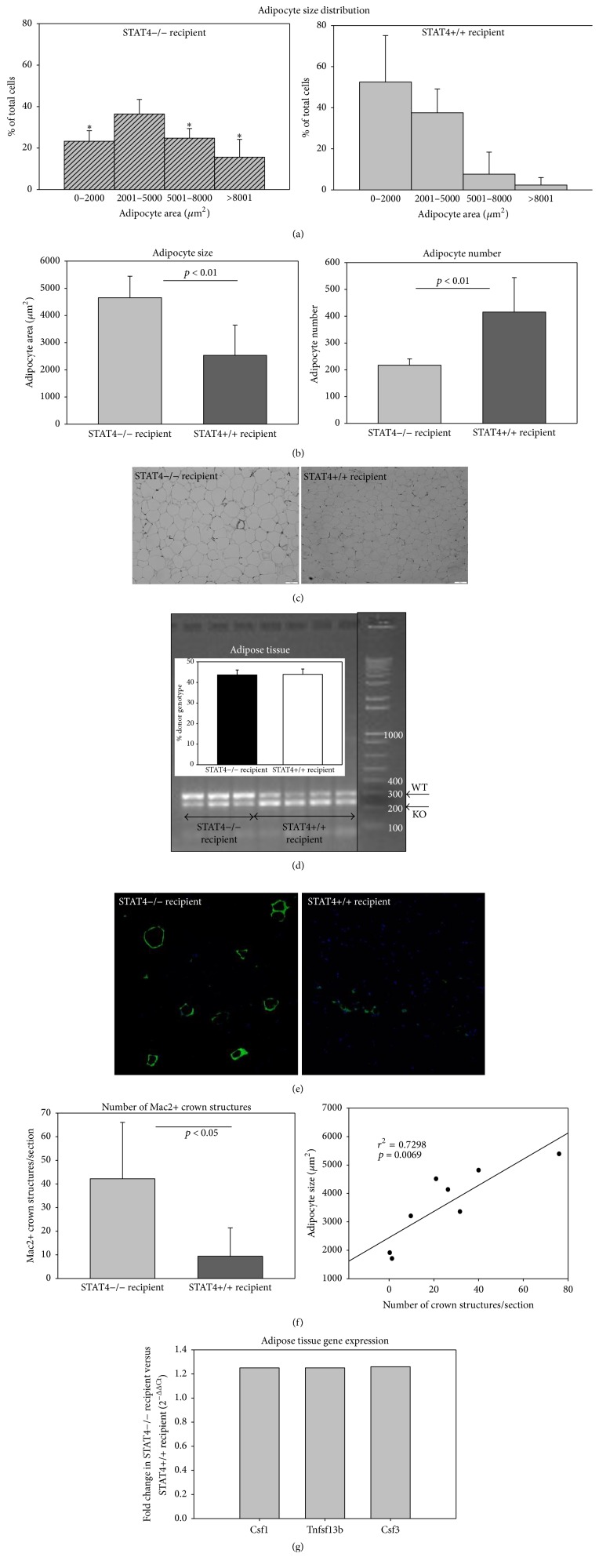
Adipose tissue morphometry and inflammation in STAT4 chimeras after 15 weeks on HFD. Adipocyte size distribution (a) and adipose tissue morphometry (b) were performed in 4 mice/group. Adipose tissue of STAT4*−/−* recipients displayed higher percentage of large adipocytes, increased average adipocyte size, and lower cellularity. Representative micrographs of epididymal adipose tissue sections are shown in (c). DNA was extracted from adipose tissue and amplified with specific primers to determine STAT4 positivity. Note that STAT4 is present in adipose tissue of both chimeras indicating significant infiltration of STAT4+/+ hematopoietic cells in the STAT4*−/−* recipients; histogram represents percentage of the donor cell genotype signal out of the total signal determined by densitometry of respective bands in agarose gels (d). Representative immunofluorescence pictures of the Mac-2 positive crown-like structures (e); morphometric analysis (f) was performed in *n* = 4 mice/group. The number of crown-like structures is significantly correlated with adipocyte size (*n* = 8 mice). Gene array containing a panel of 72 genes involved in inflammation was performed in STAT4*−/−* and STAT4+/+ recipients (*n* = 3/group) (g). Histogram shows the three significantly increased genes in STAT4*−/−* recipients. Data represents average ± SD and the null hypothesis was rejected for *p* < 0.05. ^*∗*^*p* < 0.05.

Adipose tissue inflammation is one of the key determinants of systemic insulin sensitivity. We previously showed that global STAT4 deficiency results in reduced adipocyte hypertrophy and reduced expression of inflammatory genes in adipocytes. However, it was not clear whether changes in adipocyte inflammation were the consequence of STAT4 deficiency in adipocytes or in the immune cells infiltrating the adipose tissue. The mice that received* Stat4−/−* BMC had smaller and more numerous adipocytes compared to ones that received STAT4-sufficient BMC (Figures 2(a)–2(c)). This result recapitulated the finding obtained previously in the global STAT4-deficient mice, suggesting that hematopoietic STAT4 deficiency may have the dominant effect on adipocyte phenotype. Indeed, analysis of adipose tissue from the two chimeric mouse groups shows that ~40% of the genetic signature comes from the BMC donor, indicating migration of the BM cells to the adipose tissue of the recipient (Figure 2(d)). Moreover, the number of Mac-2 positive crown-like structures (CLS) is significantly increased in the mice that received STAT4-sufficient BMC, suggesting increased inflammation in adipose tissue of* Stat4−/−* recipients compared to the* Stat4*+/+ recipients that received* Stat4−/−* BMCs (Figure 2(f)). Also, gene array analysis (PAMM150Z array, SA Bioscience) of* Stat4−/−* recipient mice adipose tissue showed a significant increase by ~1.3-fold (*p* < 0.05) of macrophage-colony stimulating factor* (Csf1)*, granulocyte-colony stimulating factor* (Csf3)*, and Tumor Necrosis Factor (Ligand) Superfamily, Member 13b (*Tnfsf13b* or BAFF) compared to* Stat4*+/+ recipients (Figure 2(g)). All three of the factors are involved in differentiation and survival of myeloid cells and B cells, respectively, suggesting that STAT4 expression in immune cells infiltrating the adipose tissue may be key for their local survival and proliferation.

**Figure 3:**
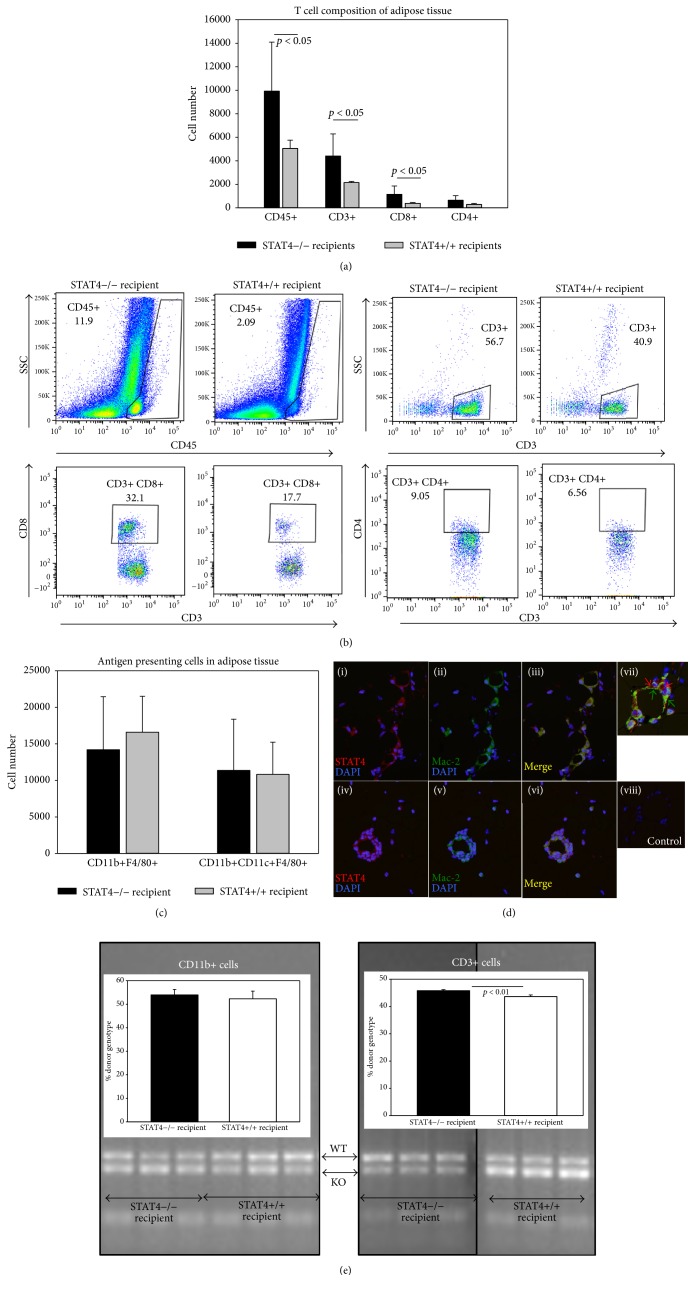
Immune cell composition of the SVF of epididymal adipose tissue of chimeric mice on HFD. (a) SVF was stained with fluorescently labeled anti-mouse CD45, CD3, CD4, and CD8 antibodies and analyzed by flow cytometry. Cell number was calculated based on the corresponding antibody positivity. To determine different T cell subsets, cells in the CD45 positive gate were analyzed. Data represent mean ± SD from *n* = 5 mice/group. (b) Representative plots showing the general strategy for the CD45+ gating and for CD3 positivity and double positivity for CD3CD4 and CD3CD8 within the CD45+ gate of each group. (c) Flow cytometry analysis of SVF stained for macrophage markers CD11b and F4/80 and the APC marker CD11c. Data represents average ± SD of 5 mice. The number of positive cells was determined within the CD45+ gate. (d) Representative immunofluorescent micrographs showing localization of STAT4 (red) and Mac-2 (green) in epididymal adipose tissue of STAT4+/+ mice (i–iii) and STAT4*−/−* mice (iv–vi); (vii) shows a crown-like structure containing some cells immunopositive for both Mac-2 and STAT4 (arrows); (viii) shows the nonimmune IgG control; magnification 200x for (i–iii), (iv–vi), and (viii); 400x for (vii). CD11b+ and CD3+ cells were isolated from adipose tissue of chimeric mice by immunoseparation and DNA was extracted and analyzed for presence of STAT4 gene following amplification with specific primers and separation on agarose gels. Band densitometry is also showed and indicates presence of both STAT4-sufficient and STAT4-deficient CD3+ and CD11b+ cells in adipose tissue of both chimeras.

Next, we examined the number and composition of the T cell population and macrophage numbers in adipose tissue of* Stat4−/−* compared to* Stat4*+/+ recipients. We found that mice that received STAT4-sufficient hematopoietic cells had significantly higher numbers of CD45+ cells, CD3+ cells, and CD3+CD8+ cells but not CD3+CD4+ cells compared to the recipients of STAT4-deficient BMC (Figures 3(a) and 3(b)). The number of CD11b+F4/80+ macrophages as well as the number of canonical proinflammatory CD11b+CD11c+F4/80+ macrophages was similar in the two mice groups (Figure 3(c)). Interestingly immunofluorescence staining showed that in both groups of mice the Mac-2 positive macrophages associated with adipose tissue crown-like structures are almost always STAT4-positive (Figure 3(d)). This may suggest a functional difference of STAT4-expressing macrophages, such as activation level and migratory properties that may have an impact on the overall local inflammatory response even in absence of a difference in cell numbers. This finding along with the STAT4 genomic signature of CD11b+ cells isolated from adipose tissue of the two chimeric groups also suggests that myeloablation does not completely remove the adipose tissue resident myeloid population (Figure 3(e)). This is also true for the CD3+ T cells, as CD3+ cells isolated from chimeric adipose tissue show a mixed STAT4 genomic signature (Figure 3(e)). Moreover, in the* Stat4−/−* recipient chimeras, the genomic signature indicates significantly higher STAT4 expression in the CD3+ cells isolated from adipose tissue, suggesting a larger number of BMC-derived CD3+ infiltrating cells compared to CD3+ BMC-derived cells from* Stat4−/−* donors. Along with the flow cytometry data (Figure 3(a)) our results suggest that the STAT4-expressing CD8+ cells are the most abundant CD3+ cells infiltrating adipose tissue in response to high fat diet.

### 3.3. Insulin Signaling in Tissues of Chimeric Mice on High Fat Diet in Response to In Vivo Insulin Challenge

To further understand the contribution of individual tissues to overall insulin sensitive profile of* Stat4*+/+ recipient mice compared to* Stat4−/−* recipient mice, we analyzed activation of insulin receptor (IR) and insulin receptor substrate-1 (IRS-1) in liver, adipose tissue, and muscle in response to an in vivo insulin bolus (Figure 6). We found increased activation of IR receptor in liver and increased IRS-1 activation in muscle of* Stat4−/−* recipients compared to* Stat4*+/+ recipients (Figure 6). This result suggests a more robust response to in vivo insulin challenge in mice that are STAT4-deficient in resident liver macrophages (possibly Kupfer cells) or in STAT4-deficient vascular cells from liver or muscle arguing for a potentially predominant beneficial role of STAT4-deficiency in cells of the nonhematopoietic compartment. Interestingly, there is no difference in adipose tissue IR activation between the two groups of chimeric mice suggesting that STAT4 in the hematopoietic compartment does not appear to be the major contributor to insulin signaling in adipose tissue in vivo (Figure 6).

### 3.4. Role of Selective STAT4-Deficiency in CD4+ and CD8+ Cells in Metabolic Profile in Response to High Dietary Fat

**Figure 4:**
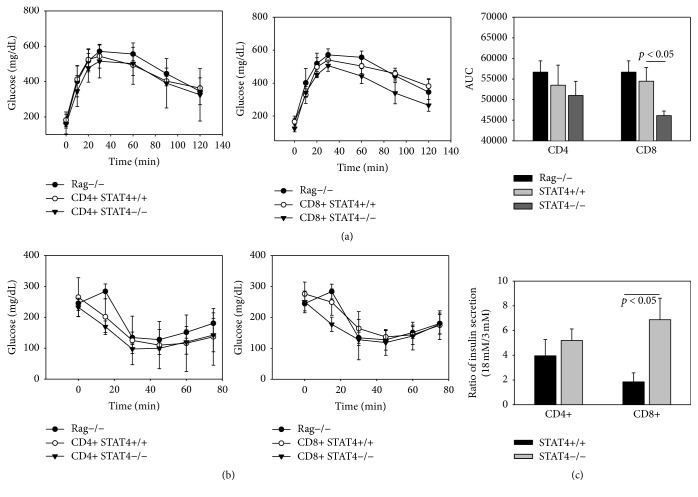
Glucose tolerance, insulin resistance, and islet insulin secretion. Rag1*−/−* controls and Rag1*−/−* mice adoptively transferred with either STAT4*−/−* CD4+ or CD8+ or with STAT4+/+ CD4+ or CD8+ cells were fed a high fat diet for 15 weeks. Intraperitoneal glucose tolerance test (GTT) (a) and insulin tolerance test (ITT) (b) were performed and area under curve (AUC) was measured for the GTT. Plotted values in the histogram are average ± SEM from *n* = 6–12 mice/group. Statistical analysis was performed using one-way ANOVA. (c) In vitro glucose stimulated insulin secretion was measured in pancreatic islets in mice after 15 weeks on HFD. Islets were cultured overnight and subsequently treated for 1 hr with 3 mM or 18 mM glucose. Insulin was measured by ELISA in the media of the islets stimulated with low or high glucose concentration. Results represent average ± SEM from *n* = 5 mice/group; the null hypothesis was rejected for a *p* value < 0.05.

To further define the role of STAT4 in lymphocyte subsets on the metabolic phenotype and adipose tissue inflammation, we adoptively transferred CD4+ or CD8+ splenocytes isolated from* Stat4−/−* mice or C57Bl6 controls into* Rag1−/−* mice. Body weight, fasting triglycerides, and total cholesterol were similar in all groups (Table 2). Also, fasting plasma glucose was similar amongst all groups. In mice that received* Stat4−/−* CD8+ cells, circulating free fatty acids were significantly lower compared to STAT4+ CD8+ recipients and compared to* Rag1−/−* controls injected with saline. Intraperitoneal GTT also showed an improved tolerance to glucose challenge in* Stat4−/−* CD8+ mice compared to* Rag1−/−* controls. The difference was borderline significant compared to mice that received STAT4+/+ CD8+ cells (Figure 4(a)). Insulin sensitivity was similar in all groups (Figure 4(b)). This result suggests potentially improved insulin secretion in response to increased glycemia. Indeed, in vitro glucose stimulated insulin secretion was significantly blunted in* Stat4*+/+ CD8+ cells but not in CD4+ cells suggesting a potentially negative effect of STAT4-sufficient CD8+ on insulin secretion (Figure 4(c)).

### 3.5. Effect of Selective STAT4-Deficiency in CD8+ and CD4+ Cells on Islet and Adipose Tissue Inflammation

**Figure 5:**
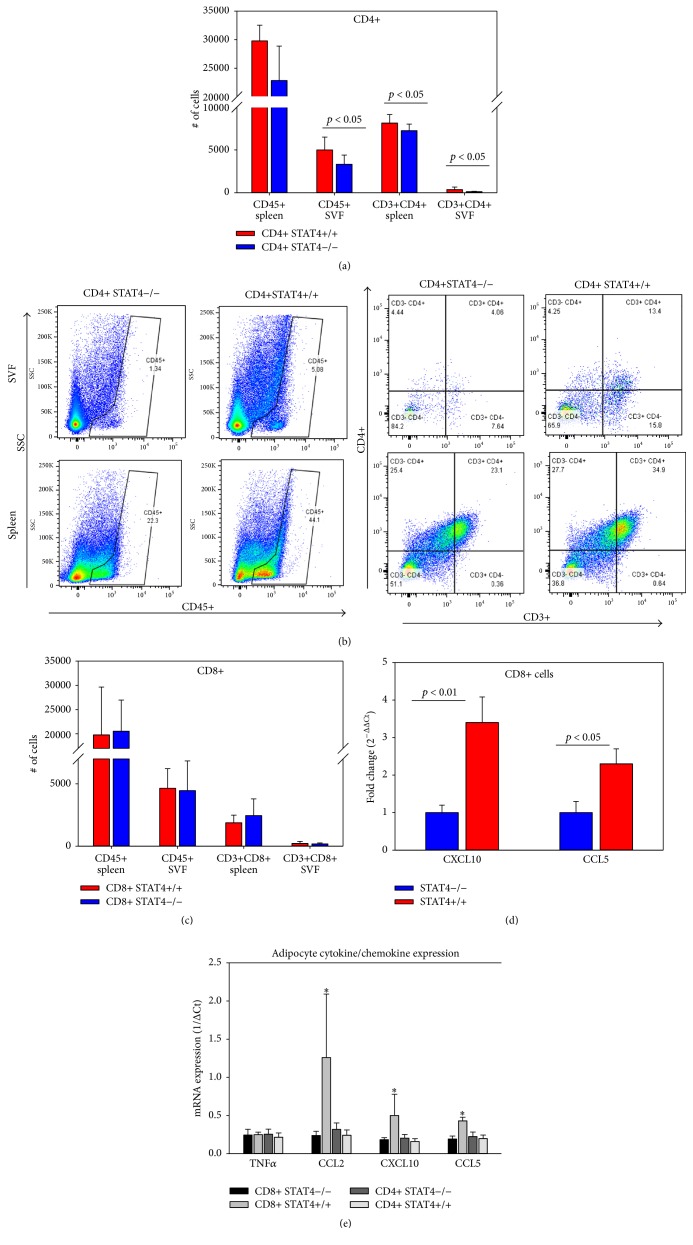
Immune composition and inflammation in epididymal adipose tissue of Rag1*−/−* mice following adoptive transfer and HFD. (a) Flow cytometry analysis of leukocyte and CD4+ T cells in adipose tissue stromal vascular fraction (SVF) and spleen of Rag1*−/−* mice following adoptive transfer with STAT4+/+ CD4+ or with STAT4*−/−* CD4+ cells. The number of cells was counted in the CD45+ gate and the double immunopositive CD3+CD4+ population was measured within the CD45+ gate. (b) Representative FACS plots illustrating the general gating strategy. (c) Same flow cytometry protocol was applied to determine the total number of leukocytes and Cd3+CD8+ positive cells in SVF and spleens of Rag1^−*/*−^ mice adoptively transferred with STAT4+/+ CD8+ or with STAT4*−/−* CD8+ cells. Histograms in (a) and (c) represent mean ± SEM from 5–7 mice/group. (d) Real-time PCR chemokine gene expression in pancreatic islets of STAT4+/+ CD8+ and STAT4*−/− *CD8+ mice after 15 weeks of HFD (*n* = 6/group). (e) Real-time PCR gene expression of cytokines and chemokines of adipocytes isolated from epididymal adipose tissue of adoptively transferred mice following 15 weeks of HFD (*n* = 6 mice/group); ^*∗*^*p* < 0.05.

We next determined the abundance of* Stat4−/−* and* Stat4*+/+ CD4+ and CD8+ cells in adipose tissue of adoptively transferred mice on high fat diet. There was no difference between the numbers of CD8+ cells in adipose tissue or spleen between the* Stat4−/−* and* Stat4*+/+ cells (Figure 5(c)). However, significantly reduced numbers of CD4+ cells were found in the spleens and adipose tissue of mice that received* Stat4−/−* CD4+ cells compared to mice that received* Stat4*+/+ CD4+ splenocytes (Figures 5(a) and 5(b)). Despite larger numbers of CD4+ cells in* Stat4*+/+ versus* Stat4−/−* recipients, there was no difference in several cytokines and chemokines in adipocytes from CD4+ mice with or without STAT4 (Figure 5(e)). However a large and significant difference was found in CCL2, CXCL10, and CCL5 in adipocytes from mice that received* Stat4*+/+ CD8+ cells compared to all of the other groups (Figure 5(e)). Similarly, islets from mice that received* Stat4*+/+ CD8+ cells showed 2.5–3.5-fold higher gene expression of the T cell cytokines CCL5 and CXCL10 (Figure 5(d)).

### 3.6. In Vitro and In Vivo Insulin Signaling in Adipose Tissue of* Rag1−/−* Mice with Selective CD8+ or CD4+ STAT4-Deficiency

**Figure 6:**
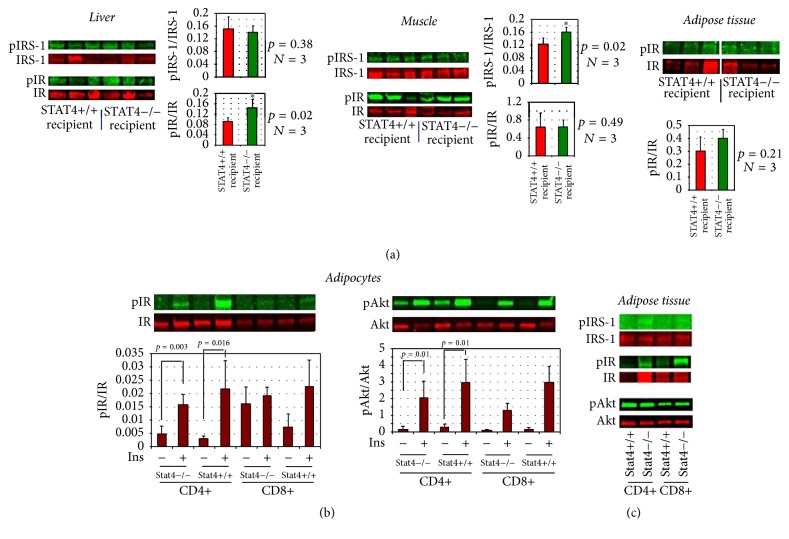
Insulin signaling in chimeric mice following bone marrow transplant and in Rag1*−/−* mice following adoptive transfer and HFD. (a) Chimeric mice following BMT. STAT4*−/−* and STAT4+/+ recipient mice (*n* = 3/group) were injected i.p. with a 10 U/kg insulin bolus and tissues were harvested after 10 minutes. Western blot analysis showed increased activation of the insulin receptor (pIR) in liver and insulin receptor substrate-1 (pIRS-1) in muscle of the STAT4*−/−* recipient mice; no differences were found between the two groups in epididymal adipose tissue. Data represents mean ± SD; ^*∗*^*p* < 0.05. ((b) and (c)) Rag1−/− mice following adoptive transfer. Adipocytes (b) were isolated from epididymal adipose tissue of different mice groups and stimulated in vitro with 5 nM insulin, for 30 min. Insulin receptor-1 and PKC (Akt) activation were measured by western blot (*n* = 3-4/group). (c) Mice were injected with an insulin bolus (10 U/kg) and euthanized after 10 minutes. Adipose tissue was harvested and processed immediately for western blot analysis. Results are representative for 2-3 mice/group.

We tested the adipocyte response to in vitro insulin challenge in mice that received STAT4-sufficient or deficient CD4+ or CD8+ splenocytes. We found that adipocytes from mice that received CD4+ cells had a robust activation of the IR and Akt that was independent of STAT4 (Figure 6(b)). However, mice that received CD8+ cells showed a less robust response. Also, IR and IRS-1 activation in adipose tissue in response to in vivo insulin challenge showed a more robust response in both CD4+ and CD8+ recipient mice that were STAT4-deficient (Figure 6(c)).

## 4. Discussion

T cells, macrophages, and adipocytes constitute a functional triad that undergoes significant deregulation in obesity and are responsible for obesity-associated disorders [2, 25]. STAT4 is expressed in all of the three cell types, but cell-specific roles in metabolic dysfunction are not clear. We previously showed that global STAT4 deficiency improves metabolic phenotype and reduces AT inflammation in obese mice [17].

In this study we investigated selective STAT4 deficiency in hematopoietic cell subsets as well as in cells from the nonhematopoietic compartment (adipocytes) on insulin resistance and adipose tissue inflammation in diet-induced obesity. Our results showed a distinct role of STAT4 in different subsets of hematopoietic cells on insulin sensitivity, glucose tolerance, and adipose tissue inflammation. We also determined that STAT4 deficiency in adipocytes may have an impact on insulin signaling in insulin sensitive tissues but has little effect on systemic insulin sensitivity and glucose tolerance. In a first set of experiments we compared* Stat4−/−* mice that received* Stat4*+/+ BMCs with* Stat4*+/+ littermates that received* Stat4−/−* BMCs. In both mice, the blood and BMC chimerism approached 100%. Bone marrow transplant with STAT4-deficient cells in* Stat4*+/+ littermates resulted in lower fasting glucose and improved insulin sensitivity and glucose tolerance compared to* Stat4*+/+ bone marrow cells transplanted into* Stat4−/−* mice, in the absence of a significant change in body weight (Table 1 and Figure 1). Bone marrow cells in C57Bl6 mice contain ~50% myeloid cells and ~18% lymphocytes [26]; however the high chimerism in spleen suggests that both the myeloid and T/B cells are contributing to the phenotype. Macrophages are known to substantially contribute to adipocyte metabolism and local inflammation via production of proinflammatory cytokines [27]. We found a significantly higher number of crown-like structures that were Mac-2 positive in the* Stat4−/−* mice that received* Stat4*+/+ BMC (Figure 2) indicating increased inflammation [28, 29]. Adipose tissue macrophages are important contributors to insulin resistance and STAT4 was shown to be required for M1 macrophage polarization and IFN*γ* production in response to IL12/18 [15]. In addition, adiponectin increases acute inflammation in macrophages in a STAT4-dependent manner [13] and STAT4 polymorphisms associated with increased STAT4 activation increased macrophage sensitivity to cytokines in humans with systemic lupus [30]. Altogether, this data suggests that macrophages lacking STAT4 may be less reactive and less responsive to cytokine challenge and therefore may display a milder proinflammatory response. Although the total numbers of CD11b+F4/80+ cells were not different in adipose tissue between the two groups, it is possible that STAT4-sufficient BM macrophages that migrated into AT in response to obesity were more proinflammatory compared to the* Stat4−/−* BM. Recruited macrophages may initially remove dying adipocytes but their activation towards an inflammatory phenotype soon results in cytokine production [23, 28]. Importantly we found that the vast majority of the macrophages localized in the crown structures showed STAT4-positivity (Figure 3). Interestingly, we did find chimeric CD11b+ populations in both mice groups, which indicated that irradiation did not result in eradication of CD11b+ cells in adipose tissue. Therefore, it is possible that STAT4-sufficient macrophages either resident or recruited contribute to “healthy” remodeling of the obese adipose tissue. However, the BM-derived macrophages that contain STAT4 are also subject to increased activation and produced more proinflammatory cytokines and chemokines. The mechanism may involve TLR4 activation in macrophages as it was shown that TLR4 requires STAT4 for IFN*γ* production in response to IL12 and ablation of TLR4 in the myeloid compartment prevents insulin resistance in obese mice [31]. Our finding that adipose tissue of mice that received* Stat4*+/+ BMC also express increased G-CSF and M-CSF (Figure 2(g)) supports the notion that the myeloid cells of the latter mice may undergo increased local activation once emigrated into adipose tissue or that STAT4 is required to induce the respective genes. Also, we previously showed that in diet-induced obesity mice with global STAT4 deletion have similar macrophage numbers compared to wild-type mice; however, there were a significantly increased proportion of the CD11b+F4/80+CD206+ cells, indicating a bias towards the M2 phenotype [17]. Importantly, our data also suggests that STAT4 deficiency in adipocytes exerts little effect on adipocyte hypertrophy or overall tissue inflammation and points towards a key role for STAT4 in myeloid and lymphoid cells.

Along with the myeloid cells, lymphocytes play an important physiologic and pathogenic role in adipose tissue in obesity. We found significantly lower numbers of CD45+ and CD3+ lymphocytes, following high fat feeding, in adipose tissue of mice that received* Stat4−/−* BMCs, compared to mice that received* Stat4*+/+ BMCs (Figure 3(a)). Interestingly, only the percentage and numbers of CD8+ T cells were significantly increased in the group that received* Stat4*+/+ BMCs. This finding recapitulates the data we reported recently for the global STAT4 deletion in obese C57Bl/6J mice, where STAT4-deficiency discriminately reduced the CD8+ cell numbers and migratory potential [17]. A recent report showed selective increase in the numbers of IFN*γ*+CD8+ cells, but not in the total CD8+ T cells in visceral adipose tissue of insulin resistant obese humans [32]. STAT4 is known to play a key role in IFN*γ* production and Th1 lineage development [11]. Therefore, this finding not only emphasizes the potentially dominant role of IFN*γ*-producing T cells but also suggests that STAT4 may be a key regulator of pathogenic CD8+ T cells in human obesity and insulin resistance.

In this study, we further investigated the role of STAT4 in different T cell subsets on insulin resistance and inflammation using a* Rag1−/−* model of diet-induced obesity. We found that glucose tolerance is significantly improved only in mice that received CD8+* Stat4−/−* cells but not CD8+* Stat4*+/+ cells, compared to* Rag1−/−* mice. No differences were found between mice that received CD4+ STAT4-deficient or STAT4-sufficient cells and the* Rag1−/−* mice (Figure 4). Interestingly, in vitro glucose stimulated insulin secretion showed an improved response in STAT4-deficient CD8+ mice compared to CD8+ controls and no difference was found between the islets of mice with or without STAT4-deficiency in CD4+ cells. It is possible that the reduced expression of CXCL10 and CCL5 chemokines found in the islets of mice adoptively transferred with* Stat4−/−* CD8+ cells (Figure 4) contributed to reduced infiltration of inflammatory cells within the islets. It was previously shown that* Rag1−/−* mice that received CD4+ lymphocytes by adoptive transfer showed better glucose tolerance and insulin sensitivity, but this effect was not observed for CD8+ cells [19]. In this study, we did not see an amelioration of the metabolic profile in mice adoptively transferred with CD4+ cells; however, we did show improved glucose tolerance in the* Rag1−/−* mice adoptively transferred with STAT4-deficient CD8+ cells. Although the numbers of CD8+ cells were similar regardless of STAT4-deficiency in both spleens and adipose tissue of adoptively transferred mice, chemokine gene expression was significantly increased selectively in mice that received STAT4-sufficient CD8+ cells compared to the other groups. In addition, gene expression of CCL2, CXCL10, and CCL5 was also increased in adipocytes isolated from* Rag1−/−* mice that received CD8+* Stat4*+/+ cells. It is possible that local chemokine production impairs insulin signaling in adipose tissue of mice that received CD8+* Stat4*+/+ cells. Indeed, although in vitro insulin stimulated adipocytes from mice that received either STAT4-deficient or sufficient CD4+ or CD8+ cells responded robustly by activation of PKB, in vivo insulin challenge triggered a more robust effect in mice that received* Stat4−/−* CD8+ cells compared to the other groups (Figure 6). The data point towards the selective pathogenic role of CD8+ cells that is blunted in the absence of STAT4 expression. Interestingly, a critical role for CD8+ in adipose tissue of diet-induced obesity on glucose tolerance and insulin resistance was also reported in relation to CD40-deficiency [33]. Although a direct molecular interaction was not yet reported for CD40 and STAT4, recent studies showed that amongst the susceptibility genes shared for both SLE and RA are CD40 and STAT4 [34, 35]. It is therefore possible that STAT4-deficiency may be protective by reducing the costimulatory effects of CD40 on CD8+ T cells. Future studies will help to address further details of the molecular mechanisms responsible for STAT4 involvement in the pathogenic role of CD8+ cells in adipose tissue inflammation and insulin resistance.

## 5. Conclusions

Collectively, our data emphasize that STAT4 in hematopoietic cells plays a key role in adipose tissue inflammation and insulin resistance. Myeloid STAT4-deficiency clearly reduces inflammation and improves the metabolic profile even in the context of STAT4 expression in adipocytes and resident STAT4-sufficient tissue immune cells. STAT4 deficiency in CD8+ cells ameliorates insulin resistance and chemokine production in adipocytes and islets and improves *β*-cell function. This study enhances our understanding on specific cellular targets that would benefit from STAT4 inhibition in vivo. The results suggest a new potential therapeutic target to reducing adipose tissue and islet inflammation and insulin resistance in obesity.

## Supplementary Material

Supplemental Figure 1 illustrates the general experimental approach. Bone marrow transplant is shown in Protocol 1. Males, 8 weeks old STAT4-/- mice and Stat4+/+ littermate controls were sub-lethally irradiated with two 6Gy doses applied 4 hours apart and reconstituted via tail vein injections with bone marrow cells (BMC) isolated from either STAT4+/+ mice or STAT4-/- homozygotes. STAT4-/- recipients received BMCs from STAT4+/+ mice and STAT4+/+ recipients received BMCs from STAT4-/- mice. Mice were allowed to fully recover for 4 weeks and were subsequently placed on 60%kcal fat diet (HFD) for 15 weeks. Protocol 2 shows adoptive transfer in Rag1-/- mice. Male, 12 weeks old Rag1-/- mice were adoptively transferred via tail vein injection with one of the following: STAT4+/+CD4+, STAT4-/- CD4+, STAT4+/+CD8+ or STAT4-/-CD8+ cells isolated from spleens of STAT4+/+ or STAT4-/- donor mice. All mice were placed on 60%kcal fat for 15 weeks. Supplemental Figure 2 shows tissue chimerism following bone marrow transplant. Representative agarose gels show the spleen, bone marrow and blood chimerism following bone marrow transplantation. DNA was isolated from each of the tissues and amplified using specific primers to detect the deletion of the STAT4 gene. In both recipient groups the chimerism with donor cells was close to 100%. Spleens of recipient mice in both groups showed a similar >85% chimerism. Histogram shows densitometry results for % donor genotype out of total signal.

## Figures and Tables

**Table 1:** Body weight, plasma lipids, and glucose for STAT4-deficient or STAT4-sufficient mice following bone marrow transplant.

Phenotype	STAT4−/− recipient	STAT4+/+ recipient	*p* value
Body weight (g)	31.7 ± 4.23	27.5 ± 3.87	NS
Total cholesterol (mg/dL)	188.71 ± 68.51	130.7 ± 35.5	NS
Free fatty acids (nmol/L)	0.66 ± 0.1	0.77 ± 0.18	NS
Triglycerides (mg/dL)	71.13 ± 10.85	62.13 ± 15.61	NS
Glucose (mg/dL)	150.25 ± 18.39	114.4 ± 19.15	<0.01

Results represent fasting body weights and plasma values at time of sacrifice. Data is from *n* = 5–8 mice/group and represents mean ± SD. Statistical analysis was performed by unpaired Student's *t*-test and the null hypothesis was rejected for *p* < 0.05. STAT+/+ recipient represents mice with selective STAT4 deficiency in bone marrow cells (see Methods).

**Table 2:** Body weight, plasma lipids, and glucose.

	Rag control	CD4+ STAT4−/−	CD4+ STAT4+/+	CD8+ STAT4−/−	CD8+ STAT4+/+
Body weight (g)	39.3 ± 2.0	44.9 ± 3.0	38.1 ± 6.3	45.4 ± 2.9	38.9 ± 5.6
Triglycerides (mg/dL)	46.1 ± 8.5	65.2 ± 13.5	111.6 ± 54.5	40.7 ± 9.5	119.8 ± 79.2
Free FA (mEq/L)	1.3 ± 0.17	0.89 ± 0.25	1.15 ± 0.25	**0.68 **±** 0.18**^*∗*^^**#**^	1.17 ± 0.17
Cholesterol (mg/dL)	170.9 ± 16.2	162.1 ± 16.4	231.9 ± 77.8	246.5 ± 60.5	215.2 ± 30.9
Fasting glucose (mg/dL)	265.9 ± 21.8	262.7 ± 65.8	259.6 ± 51.8	280.5 ± 92.3	265.1 ± 46.8

Results represent fasting body weights and plasma values at time of sacrifice. Data is from *n* = 6–12 mice/group and represents mean ± SD. Statistical analysis was performed using one-way ANOVA and the null hypothesis was rejected for *p* < 0.05. ^*∗*^Significant compared to Rag controls (*p* < 0.01). ^#^Significant compared to CD8+STAT4+/+ mice (*p* < 0.05).
