# Supplementary material for: Key Role of STAT4 Deficiency in the Hematopoietic Compartment in Insulin Resistance and Adipose Tissue Inflammation
Source: Mediators Inflamm. 2017 Mar 16;2017:5420718. doi: 10.1155/2017/5420718 (PMC5376449; doi:10.1155/2017/5420718)
Supplement: Supplementary file 1 — Supplemental Figure 1 illustrates the general experimental approach. Bone marrow transplant is shown in Protocol 1. Males, 8 weeks old STAT4-/- mice and Stat4+/+ littermate controls were sub-lethally irradiated with two 6Gy doses applied 4 hours apart and reconstituted via tail vein injections with bone marrow cells (BMC) isolated from either STAT4+/+ mice or STAT4-/- homozygotes. STAT4-/- recipients received BMCs from STAT4+/+ mice and STAT4+/+ recipients received BMCs from STAT4-/- mice. Mice were allowed to fully recover for 4 weeks and were subsequently placed on 60%kcal fat diet (HFD) for 15 weeks. Protocol 2 shows adoptive transfer in Rag1-/- mice. Male, 12 weeks old Rag1-/- mice were adoptively transferred via tail vein injection with one of the following: STAT4+/+CD4+, STAT4-/- CD4+, STAT4+/+CD8+ or STAT4-/-CD8+ cells isolated from spleens of STAT4+/+ or STAT4-/- donor mice. All mice were placed on 60%kcal fat for 15 weeks. Supplemental Figure 2 shows tissue chimerism following bone marrow transplant. Representative agarose gels show the spleen, bone marrow and blood chimerism following bone marrow transplantation. DNA was isolated from each of the tissues and amplified using specific primers to detect the deletion of the STAT4 gene. In both recipient groups the chimerism with donor cells was close to 100%. Spleens of recipient mice in both groups showed a similar >85% chimerism. Histogram shows densitometry results for % donor genotype out of total signal. [file 5420718.f1.pdf]

Supplemental Figure 1

Protocol 1. Bone marrow transplant

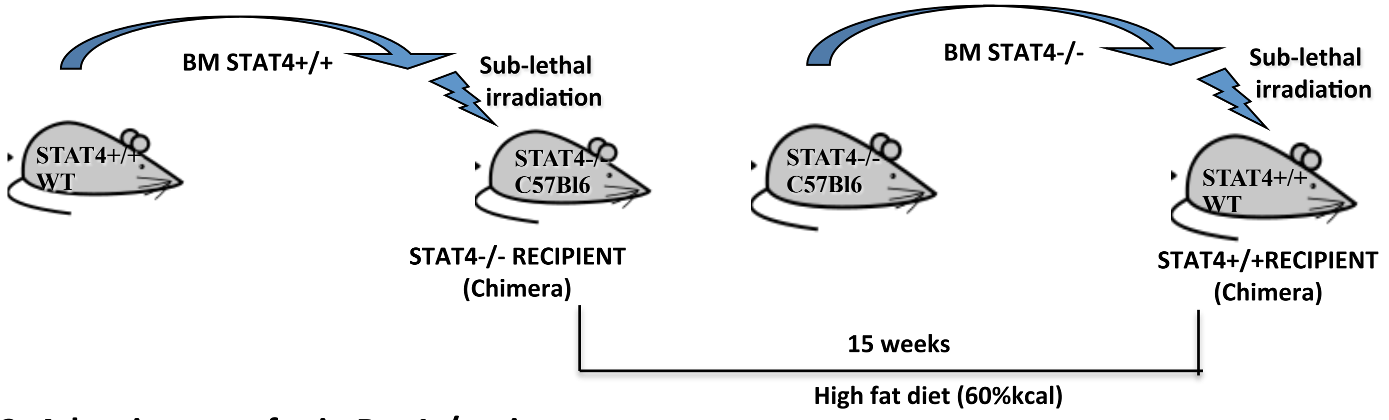

Protocol 2. Adoptive transfer in Rag1-/- mice

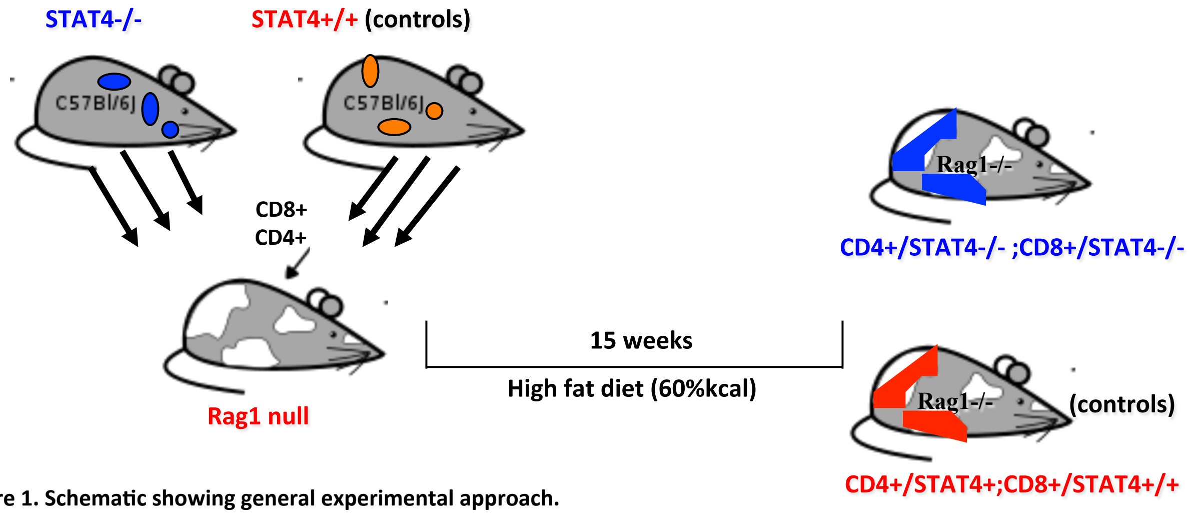

Supplemental Figure 1. Schematic showing general experimental approach.
Protocol 1 Bone marrow transplant: Males, 8 weeks old STAT4-/- mice and Stat4+/+ littermate controls were sub-lethally irradiated with two 6Gy doses applied 4 hours apart and reconstituted via tail vein injections with bone marrow cells (BMC) isolated from either STAT4+/+ mice or STAT4-/- homozygotes. **STAT4-/- recipients** received BMCs from STAT4+/+ mice and **STAT4+/+ recipients** received BMCs from STAT4-/- mice. Mice were allowed to fully recover for 4 weeks and were subsequently placed on 60%kcal fat diet (HFD) for 15 weeks.
Protocol 2 Adoptive transfer in Rag1-/- mice: Male, 12 weeks old Rag1-/- mice were adoptively transferred via tail vein injection with one of the following: STAT4+/+CD4+, STAT4-/- CD4+, STAT4+/+CD8+ or STAT4-/-CD8+ cells isolated from spleens of STAT4+/+ or STAT4-/- donor mice by immunoseparation using magnetic beads. In some experiments, Rag1-/- mice injected with saline were used as controls. All mice were placed on 60%kcal fat for 15 weeks.

Supplemental Figure 2.

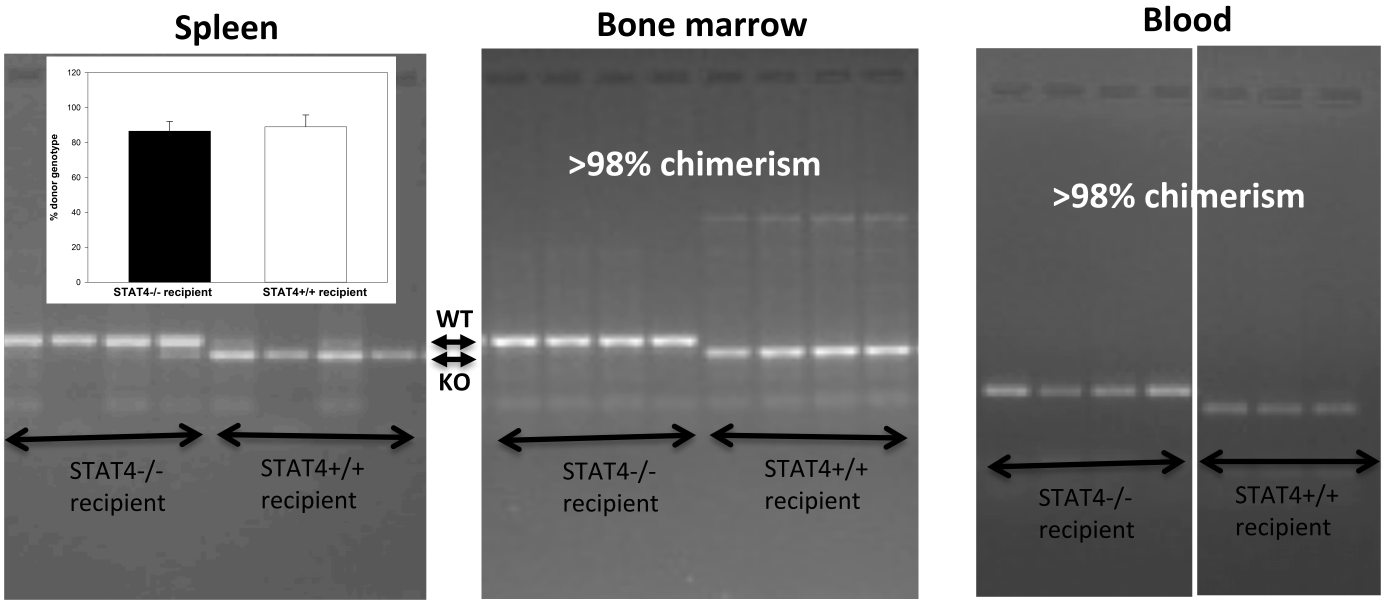

Supplemental Figure 2. Tissue chimerism following bone marrow transplant.
Representative agarose gels showing the spleen, bone marrow and blood chimerism following bone marrow transplantation. DNA was isolated from each of the tissues and amplified using specific primers to detect the deletion of the STAT4 gene. In both recipient groups the chimerism with donor cells was close to 100%. Spleens of recipient mice in both groups showed a similar >85% chimerism. Histogram shows densitometry results for % donor genotype out of total signal.
